# Supplementary material for: Antifungal susceptibility profile and local epidemiological cut-off values of Yarrowia (Candida) lipolytica: an emergent and rare opportunistic yeast
Source: Microbiol Spectr. 2023 Dec 12;12(1):e03203-23. doi: 10.1128/spectrum.03203-23 (PMC10783140; doi:10.1128/spectrum.03203-23)
Supplement: Tables S1 to S3 — Supplemental tables. [file spectrum.03203-23-s0001.docx]

**Supplementary Table 1.** Antifungal susceptibility testing (performed at 30°C) of isolates CGMCC 2.3222, CGMCC 2.1556, and CGMCC 2.1502.

|  | Anidulafungin | Micafungin | Caspofungin | 5-flucytosine | Posaconazole | Voriconazole | Itraconazole | Fluconazole | Amphotericin B |
| --- | --- | --- | --- | --- | --- | --- | --- | --- | --- |
| CGMCC 2.3222 | 0.06 | 0.12 | 0.12 | >64 | 1 | 0.12 | 0.5 | 16 | 0.5 |
| CGMCC 2.1502 | 0.12 | 0.12 | 0.25 | >64 | 0.5 | 0.5 | 0.25 | 32 | 0.5 |
| CGMCC 2.1556 | 0.06 | 0.12 | 0.25 | 2 | 0.25 | 0.06 | 0.25 | 4 | 0.5 |

Note: Sensititre YeastOne™ YO10 (Thermo Scientific, USA) was used for the antifungal susceptibility testing.

**Supplement Table 2.** *In vitro* antifungal susceptibility testing of 19 non-clinical isolates of *Yarrowia lipolytica* as determined by different methods.

|  | | Anidulafungin | Micafungin | Caspofungin | 5-flucytosine | Posaconazole | Voriconazole | Itraconazole | Fluconazole | Amphotericin B |
| --- | --- | --- | --- | --- | --- | --- | --- | --- | --- | --- |
| BMD | Range | 0.06-0.25 | 0.12-0.5 | 0.12-0.5 | 0.25 - 8 | 0.12-2 | 0.03-0.5 | 0.25-0.5 | 1-4‑ | 0.5-2 |
|  | MIC50 | 0.12 | 0.5 | 0.25 | 4 | 0.25 | 0.06 | 0.25 | 4 | 1 |
|  | MIC90 | 0.25 | 0.5 | 0.5 | 8 | 1 | 0.25 | 0.5 | 4 | 1 |
|  | GM | 0.132 | 0.333 | 0.191 | 3.333 | 0.309 | 0.064 | 0.323 | 2.582 | 0.864 |
| SYO | Range | 0.06-1 | 0.06-1 | 0.06-0.5 | 0.5->64 | 0.015-1 | 0.015-0.25 | 0.03-0.5 | 0.5-8 | 0.25-2 |
|  | MIC50 | 0.12 | 0.25 | 0.25 | 32 | 0.25 | 0.06 | 0.125 | 2 | 1 |
|  | MIC90 | 0.25 | 0.5 | 0.5 | >64 | 1 | 0.125 | 0.5 | 8 | 2 |
|  | GM | 0.146 | 0.31 | 0.184 | 20.655 | 0.322 | 0.051‑ | 0.149 | 2.678 | 0.775 |
| MTS | Range | 0.032 - 1 | 0.004-0.5 | 0.125-3 | 1->32 | 0.12 - 2 | 0.003- 0.125 | 0.25 - 2 | 0.38 - 16 | 0.047 - 0.75 |
|  | MIC50 | 0.5 | 0.125 | 1 | 8 | 0.5 | 0.064 | 0.5 | 4 | 0.19 |
|  | MIC90 | 1 | 0.5 | 3 | >32 | 1.5 | 0.125 | 2 | 12 | 0.5 |
|  | GM | 0.307 | 0.122 | 1.126 | 10.363 | 0.551 | 0.049 | 0.617 | 3.815 | 0.205 |
| ATB | Range | — | — | — | 4-32 | — | 0.06-0.25 | 0.12-1 | 1-8 | 0.5-1 |
|  | MIC50 | — | — | — | 16 | — | 0.125 | 0.25 | 4 | ≤0.5 |
|  | MIC90 | — | — | — | 16 | — | 0.25 | 1 | 8 | 1 |
|  | GM |  |  |  | 9.257 |  | 0.099 | 0.231 | 2.582 | 0.558 |
| EA (%) | SYO | 100 | 100 | 100 | 47.37 | 89.47 | 94.74 | 89.47 | 100 | 100 |
|  | MTS | 89.47 | 73.68 | 36.84 | 73.68 | 84.21 | 78.95 | 100 | 100 | 52.63 |
|  | ATB | — | — | — | 78.95 | — | 100 | 100 | 100 | 100 |

Abbreviations: BMD: broth microdilution; SYO: Sensititre YeastOne™; ATB: ATB® FUNGUS 3; MTS: Liofilchem minimum inhibitory concentration (MIC) test strip; GM: geometric mean, to allow the calculation of geometric means, high off-scale MICs were raised to the next higher concentration. EA (%): percentage essential agreement (VS. BMD); “—” means no data available.

**Supplement Table 3.** Information on the isolates included in the YALI0_B05126g (*ERG11*) analysis in this study, sourced from the National Center for Biotechnology Information Sequence Read Archive Database.

|  | Sample ID | Geographic location | Source | SRA | BioSample | BioProject | Total reads |
| --- | --- | --- | --- | --- | --- | --- | --- |
| 1 | 1031_YLIP | USA | Hospital | SRR1655228 | SAMN03196996 | PRJNA267549 | 356142 |
| 2 | 1E_07 | France | Food | ERR5235135 | SAMEA7983913 | PRJEB42834 | 47148192 |
| 3 | 24II | Poland | Environment | ERS5671210 | SAMEA7983926 | PRJEB42834 | 19127366 |
| 4 | BBE1730 | China | None | SRR9616296 | SAMN12166093 | PRJNA551788 | 136709064 |
| 5 | BBE1735 | China | None | SRR9616297 | SAMN12166094 | PRJNA551788 | 137039802 |
| 6 | BBE1737 | China | None | SRR9616295 | SAMN12166095 | PRJNA551788 | 137984406 |
| 7 | CBS_10144 | Norway | Food | ERR5235109 | SAMEA7983887 | PRJEB42834 | 33601196 |
| 8 | CBS_10150 | Norway | Food | ERR5235110 | SAMEA7983888 | PRJEB42834 | 60628582 |
| 9 | CBS_2070 | Italy | Human | ERR5235100 | SAMEA7983878 | PRJEB42834 | 50954220 |
| 10 | CBS_2787 | Germany | Human | ERP126754 | SAMEA7983880 | PRJEB42834 | 46546244 |
| 11 | CBS_5570 | Argentina | Human | ERR5235103 | SAMEA7983881 | PRJEB42834 | 51118244 |
| 12 | CBS_5589 | Argentina | Human | ERR5235141 | SAMEA7983918 | PRJEB42834 | 50513430 |
| 13 | CBS_599 | Netherlands | Food | ERR5235138 | SAMEA7983916 | PRJEB42834 | 31645128 |
| 14 | CBS_6012_50a | Denmark | Hospital | ERR5235143 | SAMEA7983919 | PRJEB42834 | 8912568 |
| 15 | CBS_6012_50b | Denmark | Hospital | ERR5235142 | SAMEA7983919 | PRJEB42834 | 12976862 |
| 16 | CBS_6124.2 | USA | Industry | ERR5235137 | SAMEA7983915 | PRJEB42834 | 50985066 |
| 17 | CBS_6125 | USA | Industry | ERR5235104 | SAMEA7983882 | PRJEB42834 | 52862718 |
| 18 | CBS_6317 | USA | Food | ERR5235105 | SAMEA7983883 | PRJEB42834 | 51988974 |
| 19 | CBS_7033 | Japan | Environment | ERR5235147 | SAMEA7983922 | PRJEB42834 | 14531054 |
| 20 | CBS_7133 | Germany | Human | ERR5235107 | SAMEA7983885 | PRJEB42834 | 45266426 |
| 21 | CBS_8108 | None | Industry | ERR5235148 | SAMEA7983923 | PRJEB42834 | 53973488 |
| 22 | CLIB_202 | Netherlands | Environment | ERR5235111 | SAMEA7983889 | PRJEB42834 | 37249084 |
| 23 | CLIB_703 | Japan | Environment | ERR5235113 | SAMEA7983891 | PRJEB42834 | 25481944 |
| 24 | CLIB_791 | France | Food | ERR5235114 | SAMEA7983892 | PRJEB42834 | 20983008 |
| 25 | CLIB_879 | Spain | Food | ERR5235115 | SAMEA7983893 | PRJEB42834 | 18129276 |
| 26 | CNRMA13.545 | France | Human | ERR5235116 | SAMEA7983894 | PRJEB42834 | 13964632 |
| 27 | CNRMA14.154 | France | Human | ERR5235117 | SAMEA7983895 | PRJEB42834 | 15211500 |
| 28 | CNRMA7.320 | France | Human | ERR5235118 | SAMEA7983896 | PRJEB42834 | 25713620 |
| 29 | DBVPG_3070 | Israel | Food | ERR5235119 | SAMEA7983897 | PRJEB42834 | 13122822 |
| 30 | DBVPG_3219 | Netherlands | Food | ERR5235120 | SAMEA7983898 | PRJEB42834 | 12136956 |
| 31 | DBVPG_3374 | Italy | Environment | ERR5235121 | SAMEA7983899 | PRJEB42834 | 14949478 |
| 32 | DBVPG_4400 | Italy | Food | ERR5235122 | SAMEA7983900 | PRJEB42834 | 25862430 |
| 33 | DBVPG_4557 | Italy | Industry | ERR5235123 | SAMEA7983901 | PRJEB42834 | 28156260 |
| 34 | DBVPG_5851 | Algeria | Environment | ERR5235124 | SAMEA7983902 | PRJEB42834 | 21846944 |
| 35 | DBVPG_6868 | Brazil | Human | ERR5235125 | SAMEA7983903 | PRJEB42834 | 14584598 |
| 36 | F1-1 | China | None | SRR8305723 | SAMN10577514 | PRJNA509301 | 20556285 |
| 37 | H222 | Germany | Environment | SRR6820826 | SAMN08667773 | PRJNA437435 | 56364306 |
| 38 | IBT_446 | Denmark | Food | SRR6820828 | SAMN08667771 | PRJNA437435 | 18644802 |
| 39 | INAG_33250 | France | Industry | ERR5235158 | SAMEA7983931 | PRJEB42834 | 11270764 |
| 40 | JII1c | Poland | Food | ERR5235151 | SAMEA7983925 | PRJEB42834 | 29744802 |
| 41 | K1-1 | China | None | SRR8305721 | SAMN10577516 | PRJNA509301 | 21720013 |
| 42 | K2 | China | None | SRR8305725 | SAMN10577518 | PRJNA509301 | 21797612 |
| 43 | LGS01.2 | Mexico | Industry | ERR5235126 | SAMEA7983904 | PRJEB42834 | 20802674 |
| 44 | LGS06.1 | Mexico | Food | ERR5235155 | SAMEA7983928 | PRJEB42834 | 27788280 |
| 45 | NCIM_3589 | India | Environment | ERR5235156 | SAMEA7983929 | PRJEB42834 | 11455628 |
| 46 | NCYC_3271 | Nigeria | Environment | ERR5235127 | SAMEA7983905 | PRJEB42834 | 8365666 |
| 47 | NCYC_3535 | United Kingdom | Industry | ERR5235128 | SAMEA7983906 | PRJEB42834 | 11110596 |
| 48 | NCYC_3727 | United Kingdom | Industry | ERR5235129 | SAMEA7983907 | PRJEB42834 | 17642958 |
| 49 | PII6a_57a | Poland | Food | ERR5235150 | SAMEA7983924 | PRJEB42834 | 4491892 |
| 50 | PII6a_57b | Poland | Food | ERR5235149 | SAMEA7983924 | PRJEB42834 | 6687310 |
| 51 | PYCC_4454 | Portugal | Food | ERR5235131 | SAMEA7983909 | PRJEB42834 | 19848090 |
| 52 | PYCC_4743 | Portugal | Environment | ERR5235132 | SAMEA7983910 | PRJEB42834 | 18427830 |
| 53 | PYCC_4811 | Portugal | Food | ERR5235133 | SAMEA7983911 | PRJEB42834 | 24971522 |
| 54 | R1-1 | China | None | SRR8305724 | SAMN10577515 | PRJNA509301 | 21765801 |
| 55 | R2-2 | China | None | SRR8305722 | SAMN10577517 | PRJNA509301 | 21778692 |
| 56 | Tmolitor69 | France | Animal | ERR5235157 | SAMEA7983930 | PRJEB42834 | 44744108 |
| 57 | W29_CLIB89 | France | Environment | SRR6820827 | SAMN08667772 | PRJNA437435 | 16508946 |
| 58 | Y14EU | None | Human | ERR268504 | SAMEA2163757 | PRJEB1905 | 9022946 |
